# Thermostable Tannase from *Aspergillus Niger* and Its Application in the Enzymatic Extraction of Green Tea

**DOI:** 10.3390/molecules25040952

**Published:** 2020-02-20

**Authors:** Yuan Shao, Yong-Hui Zhang, Feng Zhang, Qiu-Ming Yang, Hui-Fen Weng, Qiong Xiao, An-Feng Xiao

**Affiliations:** 1 College of Food and Biological Engineering, Jimei University, Xiamen 361021, China; sshaoyyuan@163.com (Y.S.); yhz@jmu.edu.cn (Y.-H.Z.); 15080038557@163.com (F.Z.); yangqm@jmu.edu.cn (Q.-M.Y.); wwhhffen2020@163.com (H.-F.W.); xiaohainan1113@163.com (Q.X.); 2 National R&D Center for Red Alga Processing Technology, Xiamen 361021, China; 3 Fujian Provincial Engineering Technology Research Center of Marine Functional Food, Xiamen 361021, China; 4 Xiamen Key Laboratory of Marine Functional Food, Xiamen 361021, China

**Keywords:** tannase, *Aspergillus niger*, thermostable, tea polyphenol, enzymatic extraction

## Abstract

Tannase is widely used in tea beverage processing because of its ability to catalyze the hydrolysis of hydrolysable tannins or gallic acid esters and effectively improve the quality of tea extracts through enzymatic extraction. A new thermophilic tannase was cloned from *Aspergillus niger* FJ0118 and characterized. The tannase exhibited an optimal reaction temperature of 80 °C and retained 89.6% of the initial activity after incubation at 60 °C for 2 h. The enzymatic extraction of green tea at high temperature (70 °C) for a short time (40 min) was devised on the basis of the superior thermal stability of tannase. The enzymatic reaction significantly increased the total polyphenol content of green tea extract from 137 g·kg^−1^ to 291 g·kg^−1^. The enzymatic reaction effectively degraded the ester catechins into non-ester catechins compared with the water extraction method. Results suggested that the thermally stable tannase exhibited potential applications in the enzymatic extraction of green tea beverage.

## 1. Introduction

Green tea is receiving considerable interest as a distinctively flavored beverage because of its antioxidant, anticarcinogenic, and antimutagenic properties [1,2] and is consumed in most Asian countries, especially in China and Japan. Tea polyphenols, including epigallocatechin gallate (EGCG), epigallocatechin (EGC), epicatechin gallate (ECG), epicatechin (EC), gallocatechin, gallocatechin gallate, and catechin contribute to the biological functions of tea [2]. As a nonfermented tea, green tea contains higher amounts of polyphenols than oolong and black teas [3]. About 75–80% of tea polyphenols are found in 30% of the soluble ingredients of tea [2,4], and tea extract products differ because of extraction methods and conditions.

The extraction conditions during tea beverage manufacturing aim to achieve the maximum extraction yield. Attempts have been made to improve the extractability of polyphenols and the clarity of tea by using pectinase and tannase for enzymatic extraction. Treatment with tannase alone can maximally improve the quality of black tea because of the high amounts of polyphenols in extracted solids [5]. Various cell wall-digesting enzymes have been used during green tea extraction to improve its quality in terms of aroma, flavor, taste, cold water solubility, and extraction yield [6]. However, enzymatic extraction can only be performed at low temperatures (30–40 °C) at prolonged treatment time (over 2 h) because of the limitations in the ideal temperatures for the enzymatic reaction [7]. Prolonged treatment leads to quality deterioration during extraction [8] and is inconvenient for industrial production.

A thermally stable enzyme should be developed to improve the efficiency of high-temperature extraction. Tannin acyl hydrolase (EC 3.1.1.20), also known as tannase, is an enzyme that hydrolyzes ester and phenolic carboxy bonds in tannins to form glucose and gallic acid (GA) [9]. The ability of tannase to break ester catechins eliminates protein precipitation during tea infusion, thereby improving the quality of the tea beverage [10,11]. Several studies have characterized tannase from plants, animals, and microorganisms. Jana et al. [12] characterized a thermostable, solvent-tolerant, and cytosafe tannase from *Bacillus subtilis* PAB2, which exhibits a half-life (*t_1/2_*) of 4.5 h at 60 °C. Gonçalves et al. [13] have reported that *Emericella nidulans* produces tannase that has hypertolerance to temperature and organic solvents and *t_1/2_* of about 72 h at 90 °C. However, most of the reported thermostable tannases are not of food grade. *Aspergillus niger* holds the Generally Recognized as Safe status from the Food and Drug Authority and is the primary filamentous fungi utilized for tannase production. The thermostability of tannase from *A. niger* is mainly between 30 °C and 50 °C [14,15,16]. Many studies have focused on the application of tannase in tea extract at 30–50 °C to reduce tea cream formation and improve the taste and color of green tea beverages [10,17,18]. The application of a thermally stable tannase derived from a food-grade microorganism in the enzymatic extraction of tea at high temperatures (≥ 70 °C) has not been studied yet.

In this work, *rAntan1*, a new thermophilic tannase, was cloned from *A. niger* FJ0118, expressed through a 5 L bioreactor fermentation, and purified using diethyl-aminoethyl anion exchange chromatography. The enzymatic and catalytic properties of *rAntan1* were investigated. *rAntan1* exhibited an optimal reaction temperature of 80 °C and retained 89.6% of its activity at 60 °C after 2 h. *rAntan1* was applied in the enzymatic extraction of tea given its superior thermal stability to enhance the extraction yield and quality of green tea.

## 2. Results and Discussion

### 2.1. Analysis of Bioinformatics and Cloning of A. Niger Tannase

**Figure 1:**
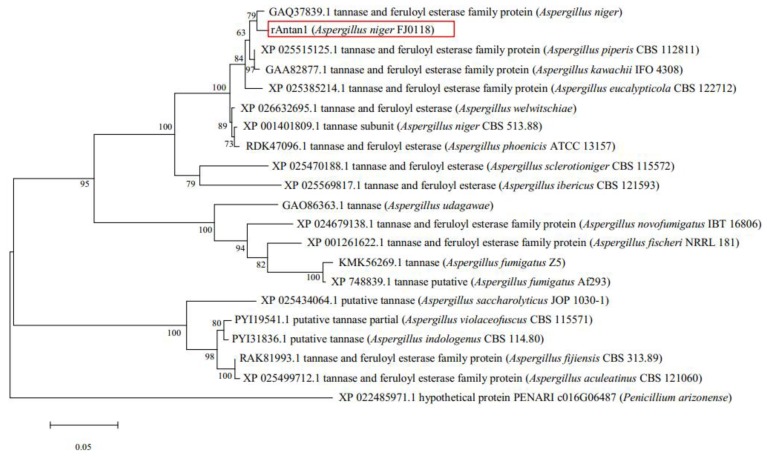
Phylogenetic relationships among known tannase family proteins. Amino acid sequence alignment was performed using ClustalW, and the phylogenetic tree was constructed using molecular evolutionary genetics analysis software version 7.0 (MEGA7). The bar represents 0.05 amino acid substitutions per site.

**Figure 2:**
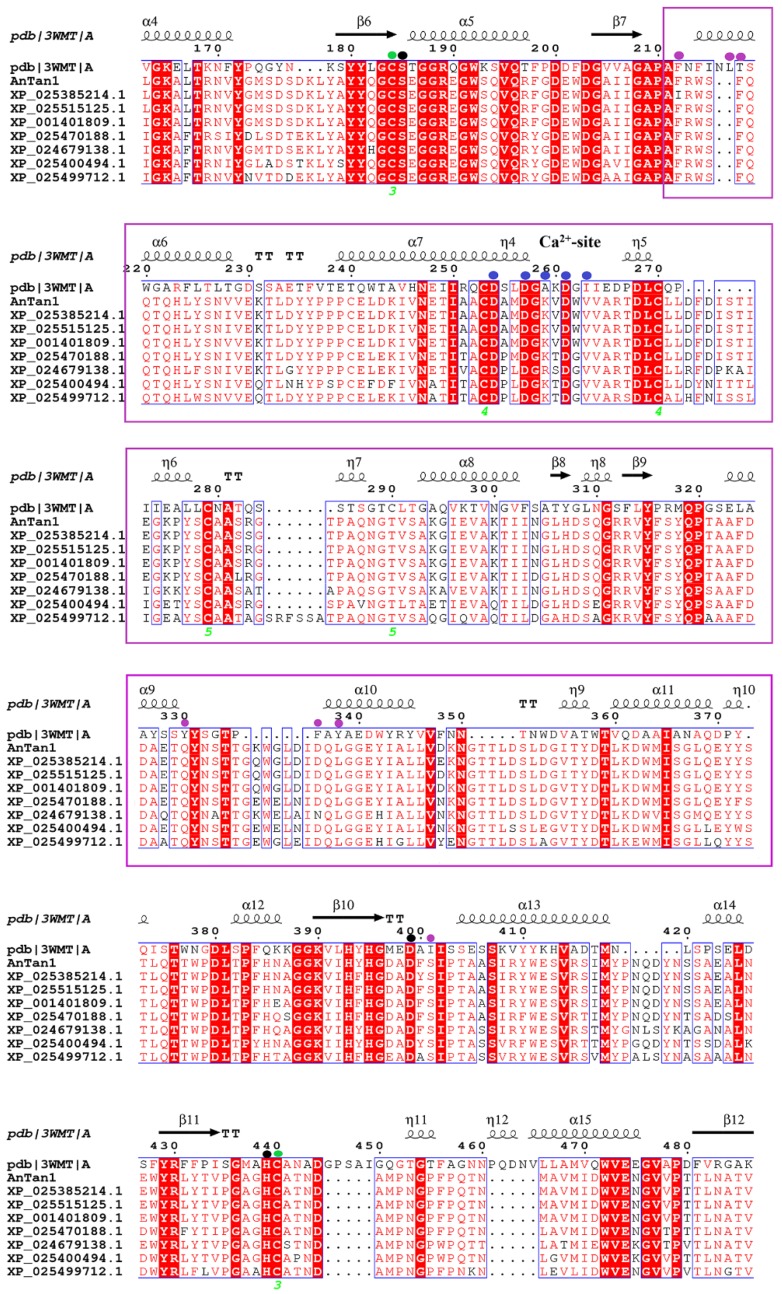
Alignment of multiple amino acid sequences of *Antan1*. The residues involved in formation of the substrate-binding pocket and the calcium-coordinating residues are indicated by black, green, magenta, and blue marks, respectively. The lid domain is boxed with magenta lines. Numbers below the sequences indicate disulfide bond pairs.

The tannase gene was amplified by PCR from *A. niger* FJ0118 genome in accordance with the sequence information of tannase (XM_001401772). The tannase gene, named *Antan1*, is 1725 bp in length without introns and codes 575 amino acids. The signal peptide of *Antan1* was predicted using the SignalP (http://www.cbs.dtu.dk/services/SignalP-4.0/) as the N-terminal 20 amino acid. The amino acid sequences were aligned using the ClustalW (http://www.ebi.ac.uk/clustalw/), which showed that the *Antan1* amino acid sequence had identity values of 98%, 95%, 91%, and 80% with tannases from *Aspergillus luchuensis* CBS 106.47 (GenBank accession number OJZ87444.1), *Aspergillus brasiliensis* CBS 101,740 (GenBank accession number OJJ71084.1), *Aspergillus carbonarius* ITEM 5010 (GenBank accession number OOF98052.1), and *Aspergillus fischeri* NRRL 181 (GenBank accession number XP_001261622.1), respectively. The phylogenetic tree was constructed for the assessment of the sequence relationship among the tannase family proteins (Figure 1). The protein functional domain of *rAntan1* (*Antan1* without signal peptide) was analyzed by comparing its sequence with the Pfam protein family database [19] (http://pfam.xfam.org/). Results indicated that amino acids 57–527 constituted a superfamily structure comprising a catalytic triad (serine–histidine–aspartic/glutamic acid). These characteristic sites were conserved in *Antan1* and predicted to be Ser206, Asp439, and His485 (Figure 2, black circle) on the basis of the multiple sequence alignment of tannases [20,21]. In addition, *rAntan1* has a “CS-D-HC motif” that is completely conserved among the biochemically distinct members of the tannase family [21]. In this CS-D-HC motif, two key residues in the catalytic triad, Ser206 and His485, are directly linked by the disulfide bonds of the adjacent cysteine residues (Cys205 and Cys486, Figure 2, green circle).

### 2.2. Tannase Production

The gene encoding *rAntan1* was successfully expressed into *Pichia pastoris* and the verified transformant *SMD*-*Antan1* was subjected to shake-flask fermentation. The *rAntan1* activity reached a maximum of 1.55 U·mL^−1^ after 144 h of induction (data not shown). Fermentation was performed using a 5 L canister to increase the yield of *rAntan1*. The maximum biomass was 309 mg·mL^−1^, and the achieved *rAntan1* activity was 390.4 U·mL^−1^ at 96 h (Supplementary Materials, Figure S1). The achieved *rAntan1* activity was 252-fold of the yield of shake flask fermentation. The enzymatic activity of *rAntan1* was higher than that of the tannases from *A. niger* Bdel4 (111.5 U·mL^−1^) [22], *Pestalotiopsis guepinii* (98.6 U·mL^−1^) [23] and *Penicillium atramentosum* (34.7 U·mL^−1^) [24], indicating the remarkable application potential of *rAntan1* in the industry.

### 2.3. Enzymatic Characteristics

**Figure 3:**
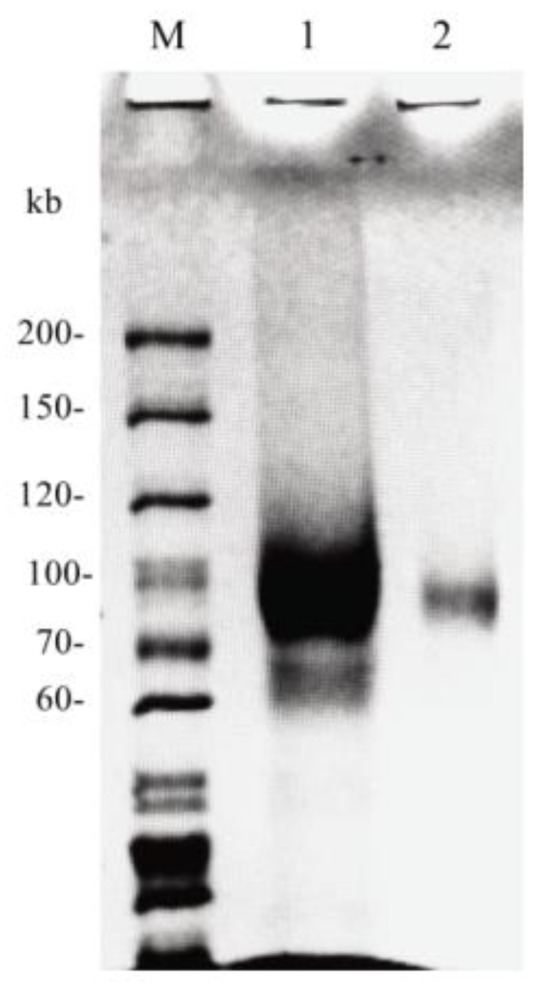
SDS–PAGE results of *rAntan1* from *A. niger* FJ0118, M: protein marker, lane 1: crude enzyme, lane 2: purified enzyme.

The molecular weight of pure *rAntan1,* as determined by sodium dodecyl sulfate (SDS)–polyacrylamide gel electrophoresis (PAGE), was 85 kDa (Figure 3), which was larger than the predicted 62.86 kDa gene product. In general, fungal tannases have a relatively high molecular weight (70–180 kDa), which is attributed to glycosylation [20,25].

#### 2.3.1. Effects of Temperature and pH on Enzymatic Activity and Stability

**Figure 4:**
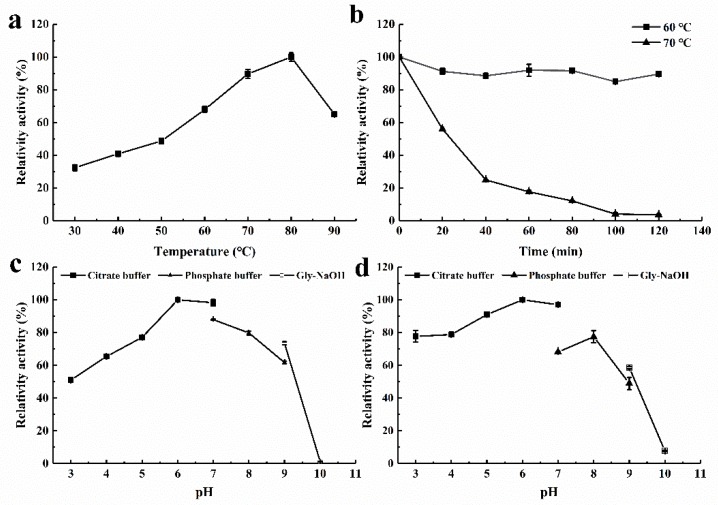
Effects of temperature and pH on the activity and the stability of *rAntan1.* (**a**) The optimal temperature for *rAntan1* was measured at pH 5.0 and different temperatures. (**b**) The thermal stability of *rAntan1* was determined after incubating at 60 °C (■), 70 °C (▲) for 120 min. (**c**) The optimal pH for *rAntan1* was determined by performing an activity assay in 50 mM citrate (pH 3.0–7.0, ■), 50 mM phosphate (pH 7.0–9.0, ▲), and 50 mM Gly-NaOH (pH 9.0–10.0, □) buffer solutions. (**d**) The pH stability of rAntan1 was determined by incubation at different pH values and 4 °C for 24 h. The residual activity was determined at the standard assay conditions.

As shown in Figure 4a, *rAntan1* activity increased with increasing temperature (30–80 °C) and exhibited optimal reaction at 80 °C. *rAntan1* retained more than 50% of its relative activity at 90 °C, showing superior heat-resistant ability. As shown in Figure 4b, *rAntan1* retained 91.9% and 89.6% of its initial activity after incubation at 60 °C for 60 and 120 min, respectively. The half-life (t_1/2_) of *rAntan1* at 60 °C was 5.4 h, suggesting its thermal stability. At 70 °C, *rAntan1* retained 17.7% of its initial activity after 60 min of incubation. In addition, *rAntan1* activity remained unchanged during incubation at 30–50 °C for 36 h (data not shown). As shown in Table 1, *rAntan1* exhibited higher optimal reaction temperature (80 °C) than most reported microbially derived tannases (20–60 °C) [13,16,20,22,26,27,28,29,30]. The tannase from *Aspergillus phoenicis* [30] retained 10% of its initial activity after incubation at 60 °C for 60 min. Compared with the tannases reported in literature, *rAntan1* is promising for thermal industrial applications.

As shown in Figure 4c, the optimal pH of *rAntan1* was 6.0, and its capacity to hydrolyze the substrate was maintained by approximately 60%–100% under a wide pH range (pH 3–8). *rAntan1* was stable at acidic to slightly basic pH conditions (3.0–8.0; Figure 4d). These results were comparable with those of the most reported fungal tannases [26,31]. However, other tannases, such as those from *E. nidulans* (pH 4.0–5.0) [13] and *Aspergillus aculeatus* (pH 5.0–6.0) [32], show a limited range of pH stability. The excellent stability of *rAntan1* at high temperatures and a wide range of pH may pave the way for carrying out biotechnological processes with minimal risk of microbial contamination with reaction efficiency.

#### 2.3.2. Effects of Metal Ions, Surfactants, Enzyme Inhibitors, and Solvents on Enzyme Activity

The effects of different metal ions on *rAntan1* activity are shown in Table 2. Most metal ions (i.e., Na^+^, Mg^2+^, Cu^2+^, Ba^2+^, Zn^2+^, Cd^2+^, Al^3+^, and Mn^2+^) inhibited the activity of *rAntan1* at 1.0 mM concentration, and Ca^2+^ had the highest inhibitory effect (32%). Cu^2+^ remarkably decreased enzymatic activity to 34% when its concentration increased to 10 mM. Heavy metal ions inhibited most tannases because the ions may have bound with sulfhydryl groups, tryptophan residues, and/or carboxyl groups at the enzyme active site [31].

As shown in Table 2, *rAntan1* was slightly inhibited by SDS, ethylene diamine tetraacetic acid (EDTA), Tween-20, and Tween-80. *β*-mercaptoethanol had a strong inhibitory effect on the activity of *rAntan1*, which was possibly because of the reduction of the disulfide bond of the active center of tannase that deactivated the enzyme [29].

The effect of organic solvents on *rAntan1* activity is shown in Table 3. At 40% concentration, the addition of *n*-hexane and cyclohexane resulted in enzyme activation by 56% and 157%, respectively, whereas dimethyl sulfoxide, *n*-butanol, *n*-propanol, and methanol showed strong enzymatic inhibition by 57%, 55%, 96%, and 77%, respectively. This result indicated that nonpolar and polar solvents can promote and inhibit, respectively, the activity of *rAntan1*. Similar reports have indicated that tannase can be activated by 60% benzene [33], whereas polar solvents, such as 20% acetone, inhibits tannase activity by 70% [13].

#### 2.3.3. Substrate Specificity

The purified *rAntan1* enzyme was incubated with different esters of phenolic acids to study its substrate spectrum. The results in Table 4 show that *rAntan1* exhibited higher activity toward natural substrates (ECG and CG) than synthetic substrates (PG). The strong degradation of ECG and CG by *rAntan1* as compared with other tannase suggests that *rAntan1* can be used for tea treatment [20].

#### 2.3.4. Kinetic Parameters

The kinetic parameters namely, the Michaelis–Menten constant (*K*_m_) and maximal velocity (*V*_max_), were calculated by the hydrolysis of PG (Supplementary Materials, Figure S2). The Lineweaver–Burk plot of *rAntan1* gave a *K*_m_ of 0.073 mM, which was lower than that of the tannases from *Penicillium herquei* (0.62 mM) [29] and *A. phoenicis* (0.6 mM) [30]. This result indicated that *rAntan1* had higher substrate affinity than these tannases. *rAntan1* exhibited *V*_max_, catalytic constant *(k*_cat_), and catalytic efficiency *(k*_cat_/*K*_m_) values of 0.29 U·mg^−1^, 1.06 × 10^3^ s^−1^ and 14.5 × 10^3^ mM^−1^ s^−1^, respectively. These values were higher than those for the tannase derived from *E. nidulans* (0.05 U·mg^−1^) [13] and *Aspergillus oryzae* (16.6 mM^−1^ s^−1^) when using PG as a substrate [28].

### 2.4. Application of Tannase in Tea Extraction

In general, the enzymatic treatment process for tea beverage can be performed in three stages, namely, tea pretreatment, extraction, and enzymatic hydrolysis [8]. Enzymatic extraction promotes extraction and improves the flavor of the extract [7], but prolonged treatment time leads to quality deterioration [8]. Several studies have reported the effects of extraction temperature, time, and method on the quality of tea infusion [34,35,36,37,38]. The tea infusion obtained using hot water showed better scavenging of free radicals than that obtained using cold water, and the extraction yield increased with increasing temperature and time. However, the active components (e.g., EGCG, EGC, and EC) decrease at temperatures higher than 80 °C because of degradation, oxidation, or epimerization [3]. Moreover, Yang et al. [39] compared the tea infusion content obtained using varying steeping temperatures (70 °C, 85 °C, and 100 °C). The tea steeped in water at 70 °C contains the highest caffeine, catechin, and GA contents.

**Figure 5:**
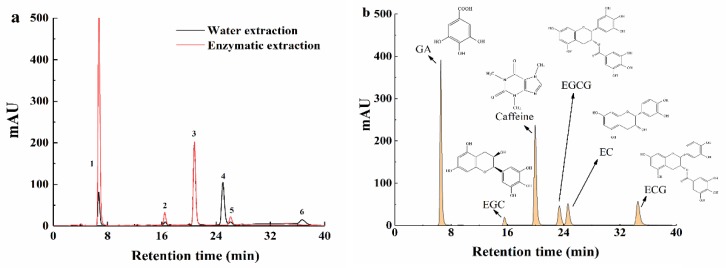
(**a**) Changes in catechins in the tea infusion after different treatment strategies. 1: GA (gallic acid), 2: EGC (epigallocatechin), 3: caffeine, 4: EGCG (epigallocatechin gallate), 5: EC (epicatechin), 6: ECG (epicatechin gallate). (**b**) Chromatograms of standards.

Thus, the excellent thermostable *rAntan1* was used in the enzyme-mediated extraction of green tea at 70 °C. The changes in catechin content in the tea infusions after different extraction methods (water and enzymatic extraction) were measured using high-performance liquid chromatography (HPLC). Figure 5a and Table 5 show that the enzymatic extraction remarkably improved the contents of non-ester catechins (EGC and EC) and decreased the content of ester catechins (EGCG and ECG). Ester catechins are the source of the bitter taste in tea infusion [40]. Thus, enzymatic extraction reduced the proportion of ester catechins in tea polyphenols, weakened the bitter taste, increased the sweet aftertaste, and improved the overall taste of the tea beverage. The amount of EGCG obtained through enzymatic extraction was almost impossible to detect (0 mg·L^−1^) compared with that obtained through water extraction. EGCG and caffeine simultaneously enhance each other’s adverse effects, such as astringency and bitterness, thereby inhibiting the development of sweet aftertaste [10]. As a nonfermented tea, green tea contains higher amounts of EGCG and ECG, which are associated with astringency and bitterness [36], compared with oolong (semifermented) and black (fully fermented) teas [40]. Therefore, enzymatic extraction can develop the sweet aftertaste of tea infusion.

In addition, a comparison of the main catechin variables and total polyphenol content showed that the enzymatic extraction of green tea improved the extract yield and remarkably changed its composition (Table 5). The polyphenol recoveries from the green tea used in the study under enzymatic and water extraction conditions were 291 and 137 g·kg^−1^, respectively. Enzymatic extraction showed an improvement by 2.1-fold. Chandini et al. [5] have reported an improvement in the polyphenol recovery through tannase-assisted extraction. This improvement may be because tannase cleaves some of the crosslinks existing between cell wall polymers that cause the degradation of plant cell wall, leading to an increase in polyphenol recovery [41]. Tea is valued for its polyphenols, which exhibit positive physiological and pharmacological effects, such as antioxidant [5], anticarcinogenic [2], and antimutagenic effects [1]. The enzymatic extraction time used in the present study achieved a significant reduction compared with other reports [5,6]. Preliminary results indicated that enzymatic extraction at high temperature is a promising approach to improve the quality of green tea extracts during the industrial production of tea beverages.

## 3. Materials and Methods

### 3.1. Microorganism

*A. niger* FJ0118 was purchased from the China Center for Type Culture Collection. *Escherichia coli* DH5α and *P. pastoris* strain SMD1168 were sourced from the Key Laboratory of Food Microbiology and Enzyme Engineering of Fujian Province (Jimei University, Xiamen, China). The extracellular expression vector pPIC9K was purchased from TaKaRa Bio (Dalian, China). Green tea (Xinyang Maojian, China) was purchased from a local market (Xiamen, China). The catechin standards (EGC, EGCG, ECG, and EC), GA, and caffeine were bought from Chengdu Biopurify Phytochemicals, Ltd. (Chengdu, China). All other reagents were of analytical grade and purchased from Sinopharm Chemical Reagent Co., Ltd. (Shanghai, China).

### 3.2. Construction of Plasmid

Primers Tan-F (5′-ATGCGCTCACCCACTCGAGTTTCC-3′) and Tan-R (5′- CTAGTACACAGGCATGGGAACCGCA-3′) were designed and compounded in accordance with the tannase gene sequence from *A. niger* CBS 513.88 (NCBI accession number XM_001401772) for the amplification of the tannase gene with the genomic DNA of *A. niger* FJ0118. The purified polymerase chain reaction (PCR) products were cloned into the pMD19-T vector (TaKaRa, Dalian, China), which constructed the plasmid pMD-Tan. The ligation mixture was transformed into *E. coli* DH5α. Positive clones were selected and confirmed by sequencing. For removing a signal peptide, Tan-F2 (5′-ACGACTCCTAGGGGGACTCCTTCCACGTTGGCGGA-3′; the AvrII site is underlined) and Tan-R2 (5′-ATAAGAATGCGGCCGCCTAGTACACAGGCATGGGAACCGCAT-3′; the NotI site is underlined) were designed and compounded to amplify the tannase gene with the plasmid DNA of the pMD-Tan as the template. The purified PCR products were digested with AvrII/NotI and ligated into the same enzyme-digested vector pPIC9K to generate the plasmid pPIC9K–Tan containing the tannase gene under the control of the alcohol oxidase 1 (*AOX1*) gene promoter. The recombinant plasmid pPIC9K–Tan was linearized with SalI and transformed into *P. pastoris* SMD1168 on the basis of the *P. pastoris* transformation protocol (Thermo Fisher Scientific, shanghai, China).

### 3.3. Production of Tannase

Shake flask fermentation was performed using buffered glycerol and buffered methanol complex media, as described by [20] to produce *rAntan1*.

The 5 L scale fermentation was performed in three phases:

(A) Glycerol fed-batch phase: Glycerol was added in batches in accordance with the change in dissolved oxygen until the biomass reached 180 mg·mL^−1^.

(B) Starvation phase: In this stage, no carbon source was added so that cell starvation for 30 min was ensured and the utilization of methanol by cells was not affected.

(C) Methanol fed-batch phase: Methanol containing 1.2% PTM1 was added at 1.5 mL h^−1^ L^−1^ during the first 12 h and increased to 3 mL h^−1^ L^−1^. This phase lasted for 96 h. The biomass and enzyme activity were measured at 8 h interval over the whole induction period.

### 3.4. Activity Assay of Tannase

*rAntan1* activity was measured by examining the production of the GA released from propyl gallate. The amount of GA was estimated through a spectrophotometric method based on the formation of chromogen between GA and rhodamine [42]. The absorbance was recorded against the inactivated enzyme blank at 520 nm. One unit of enzyme activity was defined as the amount of enzyme required to produce 1 μmol of GA per minute at assay conditions.

### 3.5. Enzyme Purification

The crude enzyme was collected from the 5 L scale fermentation by centrifugation at 4000× *g* and 4 °C for 15 min. The supernatant was filtered through a 0.22 μm nylon membrane and applied to 1.6 cm × 20 cm DEAE–Sepharose fast flow column equilibrated with citrate buffer (10 mmol·L^−1^, pH 5.0). The enzyme was eluted with a stepwise increase in NaCl (0.05 and 0.1 M) in the same buffer at a flow rate of 1 mL·min^−1^. The fractions with tannase activities were combined and concentrated using a 10-kDa ultrafiltration membrane for SDS–PAGE.

### 3.6. Characterization of Tannase

#### 3.6.1. Effects of Temperature and pH

The enzyme reaction was carried out within the temperature range of 30–90 °C to determine the optimum temperature. The thermal stability was investigated by treating *rAntan1* at various temperatures (30–70 °C) for 20, 40, 60, 80, 100, and 120 min. The optimum pH was studied by performing the enzymatic reaction in citrate (0.05 M, pH 3.0–7.0), sodium phosphate (0.05 M, pH 7.0–9.0), and glycine-NaOH (0.05 M, pH 9.0–10.0) buffer solutions. The pH stability of the *rAntan1* was determined by treating the enzyme in the same buffers at 4 °C for 24 h. The residual activity was detected under standard assay conditions and expressed as percentage of the initial activity.

#### 3.6.2. Effect of Metal Ions and Other Additives on Enzyme Activity

*rAntan1* was treated with metal ions (Na^+^, Mg^2+^, Fe^3+^, Cu^2+^, Ba^2+^, Zn^2+^, Cd^2+^, Ca^2+^, Mn^2+^, K^+^, and Al^3+^; 1 and 10 mM), surfactants (Tween-20, Tween-80, Triton X-100, SDS, and *β*-mercaptoethanol; 0.1% and 1% [*v*/*v*]), and metal chelators (EDTA; 1 and 10 mM) at 30 °C for 60 min. The enzyme activity without additives under the same measurement was determined as 100%.

#### 3.6.3. Effects of Organic Solvents on Enzyme Activity

The effects of organic solvents including dimethyl sulfoxide, hexane, butanol, cyclohexane, propanol, isoamyl alcohol, benzene, methanol, and chloroform, on *rAntan1* activity were estimated. The enzyme solution was treated at 30 °C for 60 min by using organic solvents at final concentrations of 20%, 40%, and 60%. The residual activity was detected under standard assay conditions and expressed as percentage of the initial activity.

#### 3.6.4. Substrate Specificity and Kinetic Constants of Tannase

The hydrolytic activity of *rAntan1* on EGCG, TA, PG, ECG, and CG was analyzed at a concentration of 0.01 M.

The values of the kinetic parameters, namely, *K*_m_, *V*_max_, *k*_cat_, and *k*_cat_/*K*_m_ were estimated using PG (0.05–1.0 M) as substrate at standard assay condition. The calculation of kinetic parameters was based on the Lineweaver–Burk plots [43].

### 3.7. Application of Tannase

#### 3.7.1. Enzymatic Extraction of Green Tea

Ground green tea leaves (40 mesh, 2 g) were steeped in water (leaf/water ratio of 1:50 [w/v]) and added to *rAntan1* (10 U·g^−1^ green tea). The mixture was incubated in a closed system maintained at 70 °C for 40 min for tea extraction and catechin degradation. The enzyme reaction was inactivated by incubating the mixture in boiling water for 10 min. The samples were filtered prior to the analyses of catechin and total polyphenol contents.

#### 3.7.2. Determination of Catechins and Caffeine by HPLC

The contents of catechins (ECG, EGC, EGCG, and EC), GA, and caffeine in the tea infusion samples were determined at 278 nm by using HPLC (Agilent Technologies, CA, USA). The samples were analyzed using a symmetry C_18_ column (3.0 mm × 250 mm, 5 μm). Elution was performed by using phases A (0.5% aqueous acetic acid solution) and B (acetonitrile). The elution was run for 45 min in a gradient as previously described [9]. The mobile phase flow rate was 0.5 mL·min^−1^.

#### 3.7.3. Total Polyphenols Content

The content of total polyphenols in the extracts was determined using the Folin–Ciocalteu method [44]. A calibration curve was prepared using GA as the standard, and the results were expressed in g of GA equivalent per kg of green tea (g·kg^−1^).

## 4. Conclusions

*rAntan1*, a novel thermostable tannase, was cloned from *A. niger* FJ0118, and its enzymatic properties were characterized. This tannase exhibited a *t_1/2_* of 5.4 h and 30 min at 60 °C and 70 °C, respectively, suggesting its remarkable thermal stability. A novel high-temperature enzymatic extraction was applied using the thermostable *rAntan1* for polyphenol recovery and ester catechin degradation during green tea processing. The enzymatic extraction at high temperatures resulted in high non-ester catechin and total polyphenol contents than the traditional water extraction strategy. The enzymatic extraction time was reduced significantly and more effectively than those in other similar studies. In conclusion, high-temperature enzymatic extraction is a promising approach for improving the quality of green tea extracts during the industrial production of tea beverages.

## Figures and Tables

**Table 1:** A summarized list of tannase properties.

Microorganism	Mw	Km (1) Tannic Acid (2) Methyl-Gallate (3) Propyl-Gallate	pH (1) Optima (2) Stability	Temperature (1) Optima (2) Stability (3) Residual Enzyme Activity at 60 °C for 1h	Ref.
*Penicillium notatum*	97 kDa, 43 kDa (Dimeric)	3.3 mM (1)	(1) 5.0 (2) 3.0–8.0	(1) 35–40 °C (2) up to 60 °C (3) 60%	Gayen and Ghosh 2013
*Emericella nidulans*	302 kDa	7.69 mM (3)	(1) 5.0 (2) 4.0–5.0	(1) 45 °C (2) 22–50 °C	Gonçalves et al. 2011
*Aspergillus oryzae*	45–80 kDa	n.s.	(1) 4.0–5.0	(1) 40 °C (2) up to 40 °C	Mizuno et al. 2014
*Aspergillus phoenicis*	120 kDa, 93 kDa (Dimeric)	1.7 mM (1), 14.3 mM (2) and 0.6 mM (3)	(1) 6.0 (2) 2.5–7.0	(1) 60 °C (2) 40–60 °C (3) 10%	Riul et al. 2013
*Penicillium herquei*	72 kDa	0.62 mM (3)	(1) 6.0	(1) 30 °C	Qiu et al. 2011
*Aspergillus niger* SH-2	60–70 kDa	n.s.	(1) 7.0	(1) 40 °C	Liu et al. 2018
*Aspergillus oryzae*	90–120 kDa	1.11 mM (2)	(1) 6.0	(1) 30–35 °C (2) up to 40 °C (3) 20%	Koseki et al. 2018
*Aspergillus niger* ATTC 16620	168 kDa	n.s.	(1) 6.0 (2) 4.0–8.0	(1) 40 °C (2) 30–40 °C	Sabu et al. 2005
*Aspergillus oryzae*	106 kDa	3.13 mM (1)	(1) 5.5 (2) 4.5–7.5	(1) 37 °C	Abdel-Naby et al. 2016
*Aspergillus niger*	85 kDa	0.073 mM (3)	(1) 6.0 (2) 3.0–8.0	(1) 80 °C (2) 30–60 °C (3) 91.9%	This work

n.s. means not available in the reports.

**Table 2:** Influence of metal ions, inhibitors, and surfactants on *rAntan1.*

		Residual Activity (%) *	
		1 mM (0.1%)	10 mM (1%)
Metal ions	Control	100 ± 3 ^h^	100 ± 2 ^h^
	Na^+^	86 ± 6 ^e,f^	82 ± 1 ^e,f^
	Mg^2+^	89 ± 5 ^f,g^	76 ± 6 ^d,e^
	Cu^2+^	74 ± 7 ^d,e^	34 ± 8 ^a^
	Ba^2+^	84 ± 7 ^e,f^	83 ± 9 ^e,f^
	Zn^2+^	85 ± 2 ^e,f^	86 ± 2 ^e,f^
	Cd^2+^	78 ± 1 ^d,e,f^	88 ± 4 ^f,g^
	Ca^2+^	67 ± 6 ^b,c,d^	75 ± 8 ^d,e^
	Mn^2+^	88 ± 7 ^f,g^	61 ± 1 ^b,c^
	K^+^	107 ± 10 ^h^	89 ± 2 ^f,g^
	Al^3+^	70 ± 4 ^c,d^	58 ± 8 ^b^
Surfactant and inhibitor	Control	100 ± 1 ^a^	100 ± 1 ^a^
	Tween-80	89 ± 0 ^b,c^	88 ± 4 ^c^
	Tween-20	86 ± 2 ^d^	98 ± 0 ^a^
	Triton X-100	92 ± 1 ^b^	88 ± 2 ^c,d^
	*β*-Mercaptoethanol	23 ± 1 ^f^	5 ± 0 ^g^
	SDS	85 ± 1 ^d^	69 ± 3 ^e^
	EDTA	89 ± 1 ^c^	91 ± 4 ^b,c^

* Means with different superscript letters within the same column are considerably different (*P* < 0.05). Tests were carried out using 1 mM and 10 mM of metal ions, EDTA and SDS; 0.1% and 1% (*v*/*v*) of surfactants.

**Table 3:** Influence of solvent on *rAntan1.*

Solvent	Residual Activity (%) *		
	20%	40%	60%
Control	100 ± 3 ^g,h^	100 ± 4 ^g,h^	100 ± 3 ^g,h^
Dimethyl sulfoxide	98 ± 1 ^g^	43 ± 1 ^c^	0 ± 0 ^a^
N-hexane	128 ± 10 ^k^	156 ± 5 ^l^	123 ± 9 ^j,k^
N-butanol	62 ± 4 ^e^	45 ± 2 ^c,d^	26 ± 0 ^b^
Cyclohexane	124 ± 13 ^j,k^	257 ± 2 ^n^	188 ± 27 ^m^
N-propanol	52 ± 3 ^c,d,e^	4 ± 2 ^a^	0 ± 0 ^a^
Isoamyl alcohol	106 ± 12 ^g,h,i^	85 ± 1 ^f^	56 ± 2 ^d,e^
Benzene	116 ± 4 ^i,j,k^	112 ± 4 ^h,I,j^	102 ± 2 ^g,h^
Methanol	52 ± 3 ^c,d,e^	23 ± 4 ^b^	11 ± 0 ^a^
Trichloromethane	86 ± 6 ^f^	85 ± 2 ^f^	85 ± 8 ^f^

* Means with different superscript letters within the same column are considerably different (*P* < 0.05).

**Table 4:** Substrate specificity of *rAntan1.*

Substrate	Tannase Activity (μmol·mL/min) *
PG	219 ± 2 ^c^
EGCG	103 ± 13 ^a^
TA	159 ± 3 ^b^
ECG	221 ± 2 ^c^
CG	231 ± 6 ^c^

* Means with different superscript letters within the same column are considerably different (*P* < 0.05). PG: propyl gallate; EGCG: epigallocatechin gallate; TA: tannic acid; ECG: epicatechin gallate; CG: catechin gallate.

**Table 5:** Characterization of green tea phenolic compounds.

Treatment	Contents of Single Phenolic Compounds (mg·L^−1^) by HPLC								Total Phenolics Contents by Folin-Ciocalteu Method (g·kg^−1^–GT *)
	GA	Caffeine	EGC	EGCG	EC	ECG	Non-ester	Ester	
Enzymatic extraction	1906 ± 24 ^a^	612 ± 8 ^a^	1951 ± 5 ^a^	0 ± 0 ^b^	878 ± 14 ^a^	0 ± 0 ^b^	2829 ^a^	0 ^b^	291 ± 5 ^a^
Water extraction	136 ± 2 ^b^	600 ± 9 ^a^	361 ± 5 ^b^	2687 ± 11 ^a^	130 ± 3 ^b^	980 ± 3 ^a^	491 ^b^	3666 ^a^	137 ± 1 ^b^

* g·kg^−1^–GT: polyphenol content in green tea (GT); different letters in the same column indicate significant difference (*P* < 0.05); EGCG: epigallocatechin gallate; EGC: epigallocatechin; ECG: epicatechin gallate; EC: epicatechin; GA: gallic acid; content of non-ester catechins = EGC + EC; Content of ester catechins = EGCG + ECG.

## Supplementary Materials

The following are available online at https://www.mdpi.com/1420-3049/25/4/952/s1, Figure S1: *rAntan1* production process in 5 L fermentation tanks. Figure S2. Lineweaver–Burk plot of *rAntan1*.
