# Supplementary material for: Thermostable Tannase from Aspergillus Niger and Its Application in the Enzymatic Extraction of Green Tea
Source: Molecules. 2020 Feb 20;25(4):952. doi: 10.3390/molecules25040952 (PMC7070470; doi:10.3390/molecules25040952)
Supplement: Supplementary file 1 [file molecules-25-00952-s001.pdf]

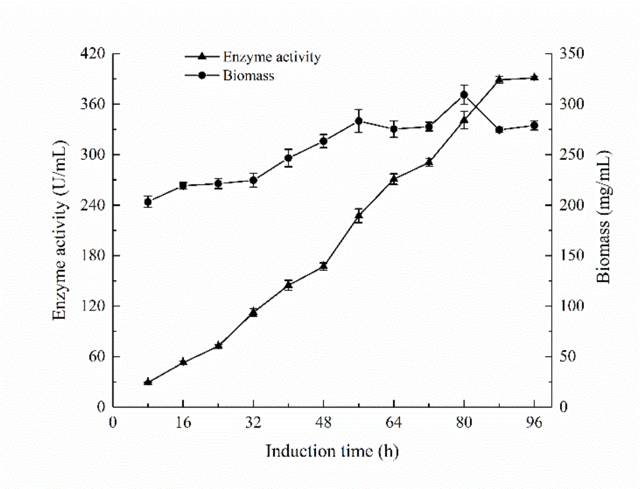

Figure S1. *rAntan1* production process in 5 L fermentation tanks.

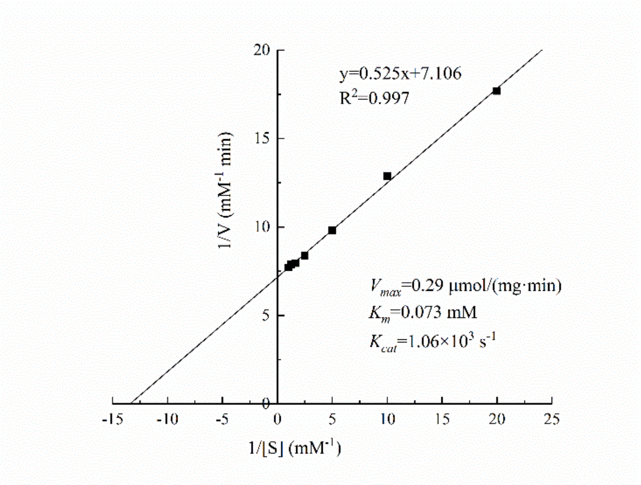

Figure S2. Lineweaver-Burk plot of *rAntan1*.
